# Evaluation of ‘vision screening’ program for three to six-year-old children in the Republic of Iran

**DOI:** 10.4103/0301-4738.57151

**Published:** 2009

**Authors:** Rajiv Khandekar, Noa Parast, Ashraf Arabi

**Affiliations:** Ophthalmologist and Epidemiologist, Low Vision Care expert, Advisor, Eye and Ear Health care, Ministry of Health, Oman; 1 Welfare Organization, Tehran, Republic of Iran.

**Keywords:** Amblyopia, childhood blindness, evaluation, Iran, vision screening

## Abstract

**Background:**

Since 1996, vision screening of three to six-year-old children is conducted every year in Iran. We present outcomes of project review held in August 2006.

**Materials and Methods:**

Kindergarten teachers examined vision by using Snellen's illiterate ‘E’ chart. They used torchlight to detect strabismus. On a repeat test, if either eye had vision <20/30, the child was referred to the optometrist. A pediatric ophthalmologist examined and managed children with strabismus or amblyopia. Provincial managers supervised the screening program. The evaluator team assessed the coverage, yield, quality and feasibility, and cost-effectiveness of vision screening, as well as magnitude of amblyopia, and its risk factors.

**Result:**

In 2005, 1.4 million (67%) children were examined in all provinces of Iran. Opticians examined 90,319 (61%) children with defective vision that were referred to them. The prevalence of uncorrected refractive error, strabismus and amblyopia was 3.82% (95% CI 3.79 – 3.85), 0.39% (95% CI 0.38 – 0.40) and 1.25% (95% CI 1.24 – 1.26) respectively. Validity test of 7,768 children had a sensitivity of 74.5% (95% CI 72.7 – 76.3) and specificity of 97.2% (95% CI 96.7 – 97.7). The cost of amblyopia screening was US $ 1.5 per child. While the cost of screening and treating one child with amblyopia was US $ 245.

**Conclusion:**

A review of the vision screening of children in Iran showed it with screening and useful exercise and had a yield of 1:21. The coverage of vision screening was low and the management of children with amblyopia, low vision and refractive error needed strengthening.

Amblyopia affects 3 to 5% of three to six-year-old children and it is a significant public health problem.[1] Early detection of amblyopia and amblyogenic factors are crucial for their successful treatment. In preschool children, strabismus, anisometropia and refractive error are associated with the higher incidence of amblyopia and hence in addition to a undetected organic conditions, they are named as amblyogenic factors.[2,3] Traditional (and modern) vision screening methods using costly equipment are usually employed.[4–8] We did not find an article on universal vision and eye screening of preschoolers in India. Kothari reported a rapid screening test for significant refractive error by using Bruckner test.[9] Each screening method has advantages and limitations. Iran has adopted vision screening using simple methods. It would be interesting to study the amblyopia screening in Iran.

The Republic of Iran is a country in the Asian subcontinent with a population of 64.5 million. Thirty per cent of this is below the age of 15. Nearly 60% reside in the urban area. The gross primary enrollment rate was 94% in 2002. The gross domestic product (GDP) in 2004 was US $ 2017. Total expenditure of the GDP on health was 6.5%. Ninety-four per cent of the population had access to the local health services in year 2000. Thus, Iran is a rapidly evolving economy with good infrastructure for education and health.[10]

The welfare organization is a separate ministry and liaises with the Ministry of Health and Universities to address different types of handicaps. For improving the quality of life of the visually disabled, vision screening of three to six-year-old children was initiated in 1996. In the first two years, it was piloted in limited areas and subsequently it was expanded to all 30 provinces of Iran. In the year 2005, the screening included 788,058 children attending kindergarten (KG). Parents brought their children who were not attending KG to a designated place in the villages for vision screening. Managers supervised the program and prepared annual progress reports. At the end of 10 years, the World Health Organization (WHO)-appointed consultant, reviewed the program of vision screening of preschoolers in Iran in August 2006 and proposed recommendations to strengthen it. We present the outcomes of this evaluation.

## Materials and Methods

We obtained written consent from the Welfare Organization of Iran and the WHO for this evaluation. Health information related to this program for the year 2005 was used for the evaluation.

**Figure 1:**
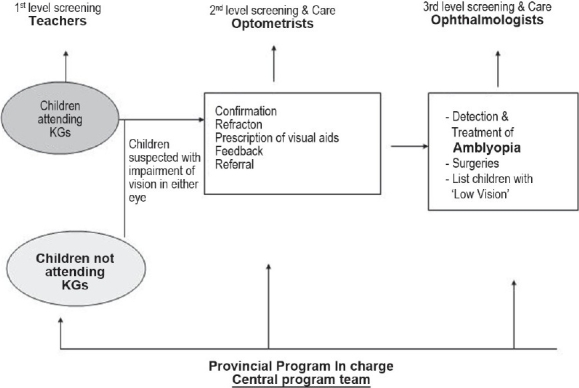
Flowchart showing levels of vision screening of three to six-year-old children in Iran

**Figure 2:**
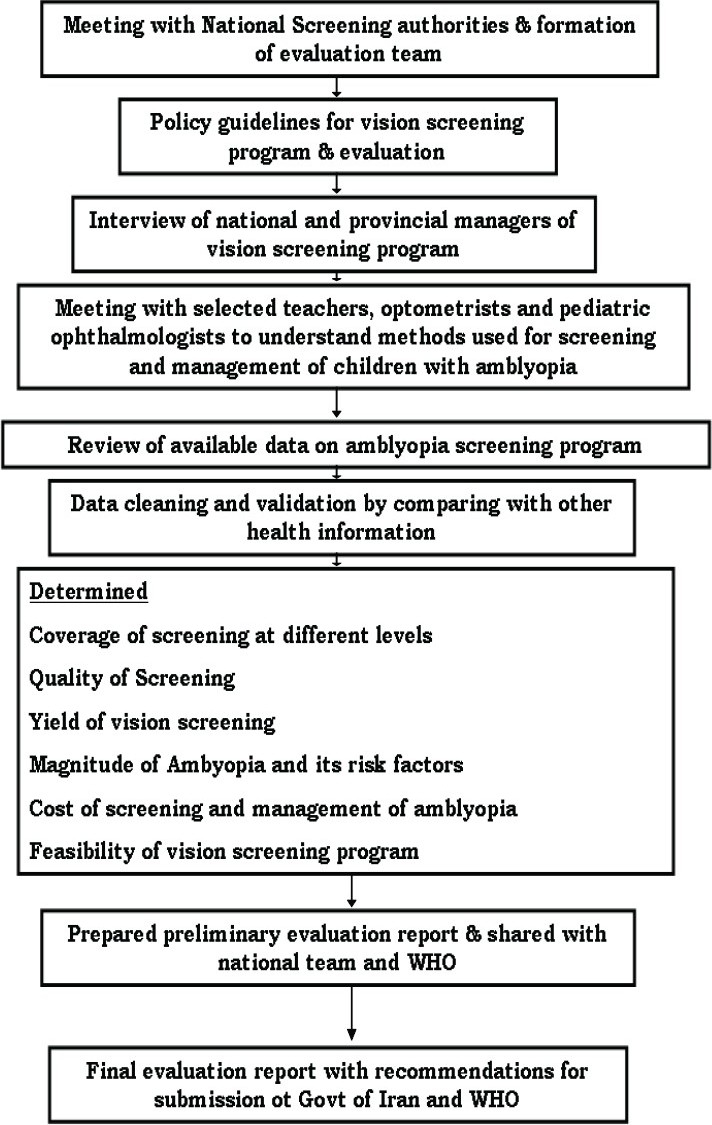
Algorithm for evaluation of vision screening program of the Republic of Iran

Ten central program staff, 30 provincial program managers, teachers, optometrists and ophthalmologists related to the program were interviewed to understand the process of the vision screening. The algorithm of the vision screening in Iran is explained in Fig. 1. The algorithm of evaluation of the amblyopia screening project is given in Fig. 2. The provincial managers briefly conduct training sessions before schools commence every year. All teachers of KG are trained in the standard method of vision screening, its interpretation and referral system. Training is carried out through lectures and practical demonstrations. Teachers screen all the children attending KG at the beginning of the academic year, examining their eyes with torchlight. This is supervised by managers to ensure a high quality of the screening. The teachers make a list of children with defective vision, communicate with parents and then refer the child to an optometrist at a nearby town. To cover children not attending KG, the program staff make announcements in villages and towns so that parents can bring their children on specified dates to the designated vision stations.

Refractive error was defined as presence of defective vision (<20/40) that could be corrected by spectacles of more than ±0.5 diopter (D). Amblyopia was defined as the blunting of vision to less than 20/30 in either eye that could not be corrected by spectacles in the absence of any other ocular pathology. Snellen's illiterate ‘E’ chart was routinely used for vision screening of school children and hence the same tools were used for preschool screening. The chart was placed six meters away from the child. Each eye was tested separately. The teachers explained procedures to students of each class and assessed in a play situation to ensure better cooperation. This assessment by teachers was labeled as first level screening. If vision was ≤20/40 in either eye, the test was repeated after two weeks. If a child was already using spectacles, the vision was noted with the spectacles. Torchlight beam was aimed between two eyes from a distance of one meter. If reflection of light was not in the center of cornea of either eye, the child was deemed on having strabismus. If vision in either eye was ≤20/40 or the child had strabismus, teachers counseled parents and referred the child to the appointed optometrist for further assessment. A list of children with defective vision from each area was prepared so that the provincial manager could follow up on the defaulters. Some parents took their children with defective vision to eye doctors instead of appointed optometrists and they were not included in calculating the coverage.

The optometrists conducted the second level of screening in their optical shops. Vision test was repeated. Cycloplegic refraction was conducted by using one drop of 0.5% tropicamide. The eye drop is repeated after 30 min if needed. Children with esotropia or excess of accommodation (noted by using fogging method) were refracted after the use of 1% atropine eye ointment applied twice for three days. Parents were informed about the possible side-effects of medication like hyperthermia, the flushing of face, glare problems and skin rashes. Subsequent to the subjective correction, spectacles were prescribed. Hyperopia was fully corrected while myopia of more than 5D was under-corrected to ensure good near-working environment. If corrected vision in both eyes had a difference of two lines, the child was referred to the pediatric ophthalmologist. Parents were counseled to ensure periodic eye checkup and incorporate changes in spectacles according to the optician's advice.

Pediatric ophthalmologists at eye units of provincial University hospitals liaised with the provincial managers of the welfare organization and examined the referred children for a detailed evaluation and managed strabismus and ocular pathologies. They provided surgical correction for congenital cataract, congenital glaucoma, strabismus and corneal opacities. Orthoptic technicians assisted pediatric ophthalmologists to treat amblyopia and strabismus. They counseled parents and ensured their cooperation with occlusion therapy for children with amblyopia.

To ensure a high quality screening, the provincial managers trained teachers, conducted review meetings and monitored their activities. A quality assessment exercise was carried out in one province. Opticians validated vision tested by the teachers. A few persons involved in the screening of children (teachers, opticians, provincial managers and parents) were interviewed to determine their acceptance of the vision screening.

The provincial manager compiled data from all three levels of vision screening and forwarded this information to the central team. Microsoft XL^®^ software was used to enter the data. The birth cohort and mortality rate in <five-year-old children were used to calculate the target population for vision screening in each province. We used Statistical Package for Social Studies (SPSS 9) to calculate frequency, percentage proportion and 95% confidence intervals of amblyopia, strabismus, refractive error and low vision. We noted population and examined sample in boys and girls of each province for estimating possible children with refractive error, amblyopia and strabismus. Accordingly, we estimated province and sex adjusted prevalence rates of these eye ailments in Iran for 2008.

Since the children with defective vision were referred both by optometrists and teachers, the coverage of children examined by ophthalmologists could not be calculated. In view of the variation in coverage of screening, the rates were adjusted at provincial levels. It was assumed that the rate of disease was similar among those examined and those suspected, but could not be examined.

Parents contributed Iranian Rial 2,000 (US $ 0.25) for the screening of a child. All children with eye ailments were offered treatment at a concessional rate. The evaluation report was used to improve the vision screening initiative of Iran.

We conducted a qualitative study through the interview of 15 parents and 15 teachers. The focus of the discussion was to identify the barriers of the vision screening and compliance of medical advices.

## Results

During 2005, we used data on live births and annual <five years' mortality rates between 1999 and 2002 to calculate the target for vision screening. Thus 2,166,851 ‘three to six-year–old’ children were targeted for vision screening in 2005. Of them, 1,433,540 (66.2%) children could be screened. We further divided the coverage into children in KG and those not attending KG. In the former group the coverage of first level vision screening was 788,058/788,058 (100%) during 2005. In the second group the coverage was 645,482/1,378,793 (47%). The coverage of first level screening for the last seven years is given in Table 1. The coverage of first level screening at vision station was 26% in the Sistan Balochistan province and 98% in the Elam province.

**Table 1:** Coverage of vision screening of ‘three to six-year-old’ children by teachers in Iran by year

Year	Target	Screened	%
1999	3,247,000	917,267	28.2
2000	3,247,000	1,036,409	31.9
2001	2,736,787	1,142,682	41.8
2002	2,580,093	1,412,167	54.7
2003	2,354,438	1,406,846	59.8
2004	2,280,749	1,492,887	65.5
2005	2,166,851	1,433,540	66.2

Of the 147,065 children with defective vision and those referred to optometrists, 90,319 (61%) children were examined. Of 1,433,540 children that were examined by teachers, 34,062 children had refractive errors. The national prevalence of uncorrected refractive error was 2.37% (95% CI 3.79 – 3.85). The sex and province-adjusted prevalence of uncorrected refractive error was 3.82% (95% CI 3.79 – 3.85). Of 1,433,540 children examined, 3,596 children had strabismus. The national prevalence of strabismus was 0.25% (95% CI 0.24 – 0.26). The adjusted prevalence of strabismus was 0.39% (95% CI 0.38 – 0.40). Opticians and ophthalmologists diagnosed amblyopia in 11,127 children. Prevalence of amblyopia in three to six-year-old children in Iran was 0.78% (95% CI 0.77 – 0.79). The adjusted prevalence of amblyopia was 1.25% (95% CI 1.24 – 1.26).

To identify one child with defective vision, the field staff had to examine at least 21 children of three to six years of age in 2005. In 2003, a similar campaign detected one child with defective vision among 18 children screened for visual acuity.

All parents of children studying in KG agreed to the vision screening. Among those not attending KG, the group discussion suggested that the lack of awareness and their preoccupation with their daily life were the main underlying causes that resulted in non-response, less coverage of vision screening and dropouts for further tests. Children attending KG loved participating in the vision screening as the procedures are non-invasive and were planned in play situations. Those performing screening were rewarded financially. Hence they were very keen to conduct the vision screening exercise with sincerity.

One hundred eighty-six teachers examined 7,768 children in KG. One optometrist repeated the vision screening in all these children. The optician was not aware of the vision screening findings of the teachers. The distant vision record of the eye with worse vision was used to label the child's visual acuity status. Optometrist's findings were used as gold standard [Table 2]. A child declared to have normal vision by the teacher was less likely to have defective vision. However, a child suspected by the teacher to have defective vision, had to be confirmed by the optometrist.

**Table 2:** Quality of vision screening of ‘three to six-year-old’ children in Iran

Validity of vision screening*			Examined by optician		

			Defective vision		
			
			Present	Absent	Total
Examined by teacher	Defective vision	Present	2,152	136	2,288
		Absent	735	4,745	5,480
		Total	2,887	4,881	7,768

Sensitivity		2,152/2,887 × 100		=	74.5%
Specificity		4,745/4,881 × 100		=	97.2%
False Positives		136/2,288 × 100		=	6.1%
False Negatives		735/5,480 × 100		=	13.4%
Positive Predictive value		2,152/2,288 × 100		=	96.6%
Negative Predictive value		4,745/5480 × 100		=	86.6%

* All 7,768 children were examined by both teachers and the optician. The latter was masked about findings of vision screening by teachers

The unit salary of the staff involved in screening and monitoring the program, cost of spectacle, eye surgery and amblyopia treatment were included in calculating the cost of the vision screening program [Table 3]. The cost of screening one child of three to six years of age in Iran was Iranian Rials 15,350 (≈US $ 1.5). The welfare organization and parents were spending Iranian Rials 2,450,000 (≈US $ 245) to detect eye problems and manage a child with an eye defect. The indirect cost for the time spent by the parents and cost of transport that parents contributed to take a child to opticians and/or ophthalmologists were not included in the cost of screening.

**Table 3:** Cost of vision screening of three to six-year-old children in Iran

	Unit cost (IR)	Persons paid in 2005	Duration involved in a year	Total cost (IR)
Salary to staff				
Teacher	2000	1433540	1.5 months	2,867,080,000
Optician	9000	90319	4 months	812,871,000
Ophthalmologist	35000	6907	4 months	241,745,000
Program staff			4 months	2,760,000,000
Cost of care				
Spectacles	200000	34062	-	6,812,400,000
Eye surgery	2000000	359	-	718,000,000
Amblyopia treatment	700000	11127		7,788,900,000
Grand Total				22,000,996,000

Unit cost of screening = US $ 1.5 = Iranian Rial 15,350, Unit cost of eye care of child with amblyopia – US $ 244 = Iranian Rial 2,450,000

## Discussion

The vision screening program identifies blinding eye conditions that are amenable to cure and rehabilitation. The program for vision screening is justified by the fact that it improves the quality of life and saves disability adjusted life years (DALY). In cases where vision screening is not undertaken before six years of age, amblyopia will be detected during enrolment in a school, which may be too late for many children. Limited improvement is usually noted in older children. In addition, Tommila *et al*, found that children with uncorrected amblyopia have a higher risk of vision loss among their peers with healthy eyes later, during their adulthood.[11] Thus, amblyopia and amblyogenic factors are important health problems and vision screening before six years of age is crucial. A nationwide screening program targeting two million ‘three to six-year-old’ children every year since 1999 in a developing country (Iran) is unique. This review was therefore important. We used the international guidelines for the development of a successful screening program.[12,13] The outcomes could therefore be compared with similar programs implemented in other countries.

Vision screening was a marathon exercise in Iran. Parents, teachers and eye care providers worked together for its success. There was a steady improvement in the coverage with every year. However, the coverage among children ‘not attending KG’ was less. The coverage also varied by province. The prevalence of amblyopia, uncorrected refractive error and strabismus was 1.25%, 3.82% and 0.39% respectively. The unit cost of vision screening was US $ 1.5. To detect and manage a child with an eye defect in Iran, the organization spent US $ 245 per child. The cost sharing model (parents and government) of vision screening in Iran was perhaps the reason for its sustainability. The vision screening model of Iran although has scope for improvement, is worth replicating in other developing countries like India, Brazil, Argentina and China that have large populations.

Factors that contributed to the success of the vision screening program in Iran were cooperation among specialists and the existence of a good referral system. Further, criteria for reporting and managing a child with the presence of amblyogenic factors were clearly defined and ophthalmologists in Iran followed them.[13] Opticians provided children with spectacles at concessional rate. Orthoptic treatment, mainly penalization and occlusion were offered to the children with amblyopia. Thus vision screening carried out in Iran fulfills one of the guidelines of early detection and treatment with minimum barriers.

Although in a hospital setup of developed countries clinicians are using tools like photo screener and autorefractor, validation and compliance in first level screening often limit their use for amblyopia screening program.[1,14] They are faster in screening children and also avoid inter-observer variations. But high cost of equipment, their inability to discover high astigmatism and hyperopia effectively should be noted before replacing the traditional vision screening.[15,16]

The vision screening in Iran was very cost-effective. It was found to be a cost-effective public health activity in the past also.[17,18] The cost of screening in Iran was much less than the cost of a similar vision and orthoptic screening reported in Germany.[17] It could have been more efficient if the coverage of screening was higher in Iran. The cost-sharing model of Iran is worth noting. Parents became more involved as they contributed financially and the organizers of the program compensated the efforts of teachers, opticians and ophthalmologists.

As coverage of first level screening was low, national prevalence of amblyopia and its risk factors were adjusted. It was assumed that the magnitude would be similar among those examined and those ‘not examined’. The children not attending KG are likely to be from poor families and they are less likely to avail health services in their early childhood. Thus risk of eye defects could be higher in the ‘not examined.’ Thus estimates of eye problems could have been underestimated in our review. We also found that the children with spectacles and having normal vision were not referred for further assessment so they were not included in calculation of refractive error. Therefore, the magnitude of uncorrected refractive error could be underestimated.[19] The grades of myopia and hyperopia were also not recorded. Thus symmetrical high refractive error as amblyogenic factor[20] could not be studied. The Snellen's illiterate ‘E’ charts were used for distant vision testing. In addition near vision was not tested.

The ‘Vision group’ of the WHO has recommended switching to the ETDRS chart for more accurate vision screening. Jones *et al*, had noted that use of ‘Lea Symbols’ was very promising.[21,22] Replacing Snellen's illiterate ‘E’ chart with ‘Lea Symbols’ in Iran and including near vision test using same symbols could further improve amblyopia screening in children of this age group.

Vision screening could be a stepping stone to initiate low vision care services. Further detailed assessment of vision function like contrast sensitivity, color vision, cognitive visual functions could be added to the vision screening in selected high-risk groups of children in future. This will improve the quality of life of children with low vision in Iran and contribute to the global initiative of low vision care of children.[14]

According to the present model, a child in KG would undergo screening thrice in Iran. Chances of detection of a new case with amblyopia and its risk factors are few once he/she is found with normal vision on first screening. Hence it is recommended that a child should undergo vision screening only once between three to six years. ETDRS (LOGMAR) charts with Lea's symbols would be more useful for screening three-year-old children.[22] The ultimate goal of vision screening is to reduce children with amblyopia by timely interventions by experts. Hence, it would be crucial to review reduction of amblyopia among children.

Review of the vision screening of children in Iran showed that screening was cost effective. This screening had yield of 1:21. The coverage of vision screening was low and the management of children with amblyopia, low vision and refractive error needed strengthening. Periodic quality checks are recommended.
